# Comparison of Disfluent and Ungrammatical Speech of Preadolescents with and without ASD

**DOI:** 10.1007/s10803-020-04747-2

**Published:** 2020-10-23

**Authors:** Mari Wiklund, Minna Laakso

**Affiliations:** 1 Department of Languages, University of Helsinki, Unioninkatu 40, P.O. Box 24, 00014 University of Helsinki, Helsinki, Finland; 2 Department of Psychology and Logopedics, University of Helsinki, Helsinki, Finland

**Keywords:** Asperger syndrome (AS), Autism spectrum disorder (ASD), Conversation, High-functioning autism, Speech disfluencies, Ungrammatical expressions

## Abstract

This paper analyses disfluencies and ungrammatical expressions in the speech of 11–13-year-old Finnish-speaking boys with ASD (N = 5) and with neurotypical development (N = 6). The ASD data were from authentic group therapy sessions and neurotypical data from teacher-led group discussions. The proportion of disfluencies and ungrammatical expressions was greater in the speech of participants with ASD (26.4%) than in the control group (15.5%). Furthermore, a qualitative difference was noted: The ASD group produced long, complex disfluent turns with word searches, self-repairs, false starts, fillers, prolongations, inconsistent syntactic structures and grammatical errors, whereas in the control group, the disfluencies were mainly fillers and sound prolongations. The disfluencies and ungrammatical expressions occurring in the ASD participants’ interactions also caused comprehension problems.

## Introduction

The aim of this paper is to analyse disfluencies and ungrammatical expressions in the conversational speech of 11–13-year-old Finnish-speaking boys who have a high-functioning autism spectrum disorder (ASD) in comparison to age- and gender-matched controls. The participants with ASD were all diagnosed with Asperger Syndrome (AS). Our analyses were quantitative and qualitative: We first identified and measured the duration of speech including disfluencies and ungrammatical expressions, and then analysed the impact of disfluent speaking turns on interaction using conversation analytic methods. The aim of the quantitative analysis was to compare the proportion of speech disfluencies in the ASD group with that of their age-matching control group. The aim of the qualitative analysis, in turn, was to examine the types of disfluencies and ungrammatical expressions in more detail and to observe how other participants in the interaction reacted to them.

Both speech disfluencies and ungrammatical expressions interrupt the continuous flow of speech and may thus present challenges for conversational interactions. When speech is fluent, speech production flows smoothly both in terms of sound and information (e.g., Manning 2010). There are few interruptions, and listeners can concentrate on the content of speech and are not disturbed by the manner in which the speech is produced. Disfluency refers to the interruption of on-going speech and exhibits features such as silent and filled pauses, sound prolongations, repetitions and cut-off utterances (e.g., Shriberg et al. 2001). Disfluencies have been connected to speech planning and processing, and they are relevant for listeners’ comprehension of speech (e.g.,Wiklund and Laakso 2019). Furthermore, normally fluent speakers present disfluencies, i.e., they hesitate, repeat, and interrupt their speech in order to formulate their expressions (e.g., McDougall and Duckworth 2017). These speakers also display considerable individual variation in the rate and types of disfluency features they produce. Disfluencies are phenomena that assist speakers in planning and articulating their speech and allow time for the listeners to understand what was stated. Disfluencies can also be more disturbing and can be divided into *stuttering-like disfluencies* (SLD) and *other disfluencies* (OD) (Ambrose and Yairi 1999; Byrd et al. 2012; Tumanova et al. 2014). Ambrose and Yairi (1999, p. 899) include in the category of SLD (a) part-word repetitions (“b-but,” “thi-thi-this”), (b) single-syllable word repetitions (“you,” “and and”), (c) disrhythmic phonations, such as prolongations (“mmmmy,” “cooookie”), blocks (“#toy”), and broken words (“o#pen”). The OD group, in turn, includes (d) interjections (“um”), (e) revisions/abandoned utterances (“Mom ate/Mom fixed dinner,” “I want/Hey look at that”), and (f) multisyllable/phrase repetitions (“because because,” “I want I want to go”). Disfluencies have been defined in varying ways using different terminology, and researchers have not always held similar views on what can be defined as stuttering-like and other disfluencies.

Several previous studies have demonstrated that many speakers with ASD, and especially AS, produce disfluent speech (e.g., Shriberg et al. 2001; see Table 1 that presents the main results of a number of previous studies related to disfluencies in speech of adults and school-aged children with AS or high-functioning autism, HFA). Although the definitions of disfluencies differ, the studies report that speakers with AS have been found to produce numerous stuttering-like disfluencies such as sound, syllable, or word repetitions but also atypical disfluencies such as breaks, insertions and word-final sound prolongations.

**Table 1:** Main results of previous studies on disfluencies of persons afflicted with AS and HFA

Authors	Participants	Main results
Shriberg et al. (2001)	15 male speakers with High-Functioning Autism (HFA) and 15 male speakers with Asperger syndrome (AS) compared to one another and to profiles for 53 control male speakers in the same 10 to 50 years age range	Differing from the controls, 67% of the individuals with AS and 40% of the individuals with HFA had inappropriate or non-fluent phrasing, including sound, syllables, or word repetitions and single-word revisions in more than 20% of their utterances
Lake et al. (2011)	13 adults on the autism spectrum (4 of this group were diagnosed with AS) and 13 controls	An increased number of silent pauses and disfluent repetitions in the autism group as compared with the controls. Fewer listener-oriented disfluencies such as filled pauses and revisions in the ASD group’s speech than in the control group’s speech
Scott et al. (2006)	2 young adults with AS	The speech of the informants included stuttering-like disfluencies (part-word repetitions and blocks), as well as non-stuttering-like disfluencies (phrase repetitions and interjections)
Sisskin (2006)	2 persons with AS (aged 7 and 17 years)	Both informants exhibited within-word stuttering-like disfluencies (part and whole-word repetitions and blocks) and between-word non-stuttering-like disfluencies (phrase repetitions, revisions and interjections). Stuttering-like disfluencies were either mid-syllable insertions (defined as a short exhalation resembling the production of /h/) or word-final disfluencies (repetitions in which the repetition forms a rhyme by omitting the initial consonant(s) or syllable of the target word, e.g. ‘train-ain’) not typical of developmental stuttering
Scaler Scott et al. (2014)	11 school-aged children with AS, 11 matched children who stutter, and 11 matched control children with no diagnosis	Statistically significant differences between children with AS, children who stutter and those with no diagnosis for the percentage of words containing stuttering-like disfluencies. AS participants’ speech included a larger distribution of word-final disfluencies
Wiklund and Laakso (2019)	7 school-aged (11- to 13-years-old) Finnish-speaking boys with AS	The speech of the autistic preadolescents included frequent disfluencies and morpho-syntactic problems, such as incorrect case endings, ambiguous pronominal references, grammatically incoherent syntactic structures and inaccurate tenses, which caused problems in comprehension during interaction

Two of the studies on adult speakers with AS that include a control group demonstrated that speakers with AS produce more disfluencies than the controls (Shriberg et al. 2001; Lake et al. 2011). Shriberg et al. (2001) observed that when compared to controls, significantly more participants in both the AS and HFA groups had non-fluent phrasing that included sound-, syllable-, or word-level repetitions or part-word revisions, also with multiple occurrences of these behaviours within one utterance. Lake et al. (2011) detected both quantitative and qualitative differences: Adult speakers with AS produced fewer listener-oriented disfluencies such as filled pauses and revisions and more silent pauses and repetitions that reflected the speaker’s speech processing.

Three of the studies compare the disfluencies in AS with stuttering and report both similarities and differences. Studies by Scott et al. (2006) and Sisskin (2006) presented each two cases with AS without matched controls, and Scaler Scott et al. (2014) examined a group of school-aged children with AS and age-matched control groups of children who stutter (CWS) and children with no diagnosis (ND). The results of Scaler Scott et al. (2014) reveal that the three participant groups displayed differences that were statistically significant in terms of the percentage of words that contained stuttering-like disfluencies. CWS produced a higher percentage of stuttering‐like disfluencies (41% for CWS; 21% for children with AS), while the children with AS produced a higher percentage of word-final disfluencies^1^ (5% for children with AS and 1% for CWS). Word‐final disfluencies were present in the speech sample of eight out of 11 children with AS. By comparison, word‐final disfluencies were present in four out of 11 children of the CWS group and three out of 11 children of the ND group. The speech of two young adults with AS analysed by Scott et al. (2006) included both stuttering-like disfluencies (part-word repetitions and blocks), as well as non-stuttering-like disfluencies (phrase repetitions and interjections). In Sisskin’s (2006) two case studies, the disfluencies of the participants with AS were not typical of a developmental fluency disorder.

The definition and classification of disfluencies differed in the studies presented in Table 1, but all studies found that speakers with AS frequently have disfluent speech with at least repetitions, and some studies also suggest that speakers with AS are more disfluent than the controls. Several studies also indicate that speakers with AS revise and self-repair their speech (Shriberg et al. 2001; Sisskin 2006; Wiklund and Laakso 2019), although contradictory findings have also been reported (Lake et al. 2011). Insertions (Lake et al. 2011), interjections (Scott et al. 2006; Sisskin 2006), pauses (Lake et al. 2011) and word-final prolongations (Scaler Scott et al. 2014; Sisskin 2006) have also been found to occur. Stuttering-like disfluencies (e.g. part or whole word repetititions and blocks) were observed in three studies (Scaler Scott et al. 2014; Scott et al. 2006; Sisskin 2006), but school-aged children with AS nonetheless differed from children who stutter. In short, all seven studies presented in Table 1 have had small samples ranging from 2 to 15 speakers with ASD and only three studies have presented control group data. Furthermore, only the study by Scaler Scott et al. (2014) examined preadolescents with a mean age of 11 years. Thus, additional research is needed to compare the disfluencies of preadolescents with ASD with an age-matched control group. Furthermore, as disfluency may disturb conversation in a similar manner as stuttering, we need to determine the interactional consequences for social conversation caused by the disfluent speech production of preadolescents with ASD.

Ungrammatical expressions also create an impression of disfluency in the speech of people with ASD (Wiklund and Laakso 2019). The prior study demonstrated that aspects that make the speech flow disfluent and difficult to follow are incorrect case endings, ambiguous pronominal references, grammatically incoherent syntactic structures and inaccurate tenses. Indeed, syntactic impairments have been found in individuals with ASD (Cummings 2014). For example, children with ASD may align their use of syntactic structure to that of a conversational partner (Allen et al. 2011). They also encounter difficulties in their grammatical comprehension of instructions (Saalasti et al. 2008) and tend to map verbs onto causative actions (Naigles et al. 2011). McGregor et al. (2012) concluded that only those children with ASD who do not have syntactic deficits demonstrate an age-appropriate knowledge of words. ASD children’s word learning has also been found to be compromised owing to their reduced sensitivity to the social information of gaze cues (Norbury et al. 2010). Thus, children with ASD may have more profound linguistic difficulties underlying their challenges in discourse and social interaction (see also Saalasti et al. 2008; Wiklund 2016; Wiklund and Laakso 2019).

Even though disfluencies by people with ASD have been studied before, the current analysis is methodologically innovative in that it adopts the framework of conversation analysis (CA) (see e.g. Heritage 1984). CA has already been used by researchers when conducting research with individuals with ASD (see for example *Journal of Autism and Developmental Disorders* Vol. 46, Issue 2, Feb. 2016), but a combination of CA, quantitative measures and the use of a control group in the study of disfluencies of persons with ASD is a new approach. Overall, CA is the study of recorded, naturally occurring talk-in-interaction. The aim of studying these interactions “is to discover how participants understand and respond to one another in their turns at talk, with a central focus on how sequences of actions are generated” (Hutchby and Wooffitt 2008, p. 12). Concerning atypical and asymmetric interactions (such as the therapeutic conversations of our data), in contrast to the research that focusses on individuals’ impairments, CA research has the potential of revealing participant collaboration and resources during interaction. Therefore a study adopting the methods of CA may lead to an increased understanding of the causes and interactional effects of the disfluency phenomena.

The main research objectives of this study are (1) to compare the quantity of disfluencies by preadolescents with ASD and typical development, and (2) to examine the interactional consequences of disfluencies in more detail in order to determine whether differences can be detected between the groups.

## Data and Methods

### Data and Participants

The data of this study consist of audio-visual recordings of neuropsychiatric group therapy sessions in which two groups of 11- to 13-year-old boys engage in a discussion with their therapists and each other in southern Finland in 2009 (Group A) and in 2010 (Group B). All the informants of the ASD groups are native speakers of Finnish. Group A consists of three participants and two therapists, and Group B consists of four participants and two therapists. One of the therapists is a man and the other is a woman. The male therapist is the same in both sessions; the female therapist is not. Both groups include one member who had not been diagnosed with ASD at the time the data was recorded, although according to the therapists, both individuals exhibited the same symptoms as the other members of the group. We have decided not to include these two persons without a diagnosis in our data. This means that the number of the informants in this study is five, even if the total number of participants in the sessions is seven. The five participants with ASD in the research group were assigned the codes A1 to A5, and their names have been replaced with pseudonyms in the conversational data extracts. Similarly, the control group were given codes C1 to C6, and pseudonyms. The codes and the corresponding pseudonyms are listed in Table 2.

**Table 2:** The codes and the corresponding participant pseudonyms

ASD group	Control group
A1 Harri	C1 Eero
A2 Markus	C2 Pentti
A3 Mikko	C3 Pekka
A4 Kalle	C4 Aaron
A5 Jaakko	C5 Miikka
	C6 Otto

Diagnoses of the participants were gathered as background information. All participants with ASD were diagnosed with Asperger Syndrome, and had normal IQ. Some of the participants had co-morbid diagnoses. The diagnoses are given in Table 3.

**Table 3:** Diagnoses of participants with ASD

Participant	Diagnoses
A1 Harri	Asperger syndrome
A2 Markus	Asperger syndrome, attention deficit/hyperactivity disorder, Tic disorder
A3 Mikko	Asperger syndrome, with difficulties in executive functions
A4 Kalle	Asperger syndrome, with difficulties in social interaction, attention and executive functions
A5 Jaakko	Asperger syndrome

It is noteworthy that three of the participants, A2 (Markus), A3 (Mikko) and A4 (Kalle), had attentional deficits and/or difficulties in executive functioning as co-morbid diagnoses, in addition to the Asperger diagnosis.

The duration of each session included in the data was 1 h.^2^ Two cameras were used to film these sessions. Each participant also had a microphone behind one ear. The filming and the recording were carried out by audio-visual technology professionals. The sessions began with sharing news: each participant told about what he had been doing lately, how school was going, and other related matters. After hearing a participant’s news, the others asked questions concerning what they had just heard.^3^ After this, the group discussed a predetermined theme with the help of a series of drawn pictures. In both sessions filmed for this study, the theme was bullying at school.

The control group data were taken from a 30-min-long conversation in which six 11- to 13-year-old Finnish-speaking boys talk with their female teacher. The control group data were recorded in southern Finland in 2016.^4^ Two cameras were used to film the session, and each participant had a microphone behind one ear. The filming and the recording were conducted by audio-visual technology professionals. The sessions began with sharing news, and after this, the group discussed bullying at school. The situation was not authentic, but it was constructed for the purposes of the current research project to match the therapy discussions. All the informants of the control group were neurotypical native Finnish speakers with normal IQ. None of them was bilingual.

All data were collected with the informed consent of those involved. The study was evaluated and approved by the hospital ethics committee (decision number 284/13/03/03/2009).

### Analytical Methods

Our analyses are quantitative and qualitative in that we first measured the durations of all speech extracts including disfluencies and ungrammatical expressions, and then analysed them qualitatively using conversation analytic methods (e.g., Hutchby and Wooffitt 2008). We decided to include ungrammatical expressions in this study because they also create an impression of disfluent speech and are typical of the informants in our data (Wiklund and Laakso 2019).

In the quantitative analysis, the speech analysis programme “Praat” (Boersma and Weenink 2016) was used to measure the durations of disfluent and ungrammatical extracts of speech. The percentages for the durations of disfluent and fluent speech were then compared between individuals and the groups. After that, the “Atlas.ti7” programme (ATLAS.ti Scientific Software Development GmbH 2016) was used to classify and to code the occurrences of disfluent versus ungrammatical speech. For the coding, the speaking turns for each of the preadolescent participants in the ASD and control groups were identified and the turns were subsequently classified as turns including disfluencies, turns including ungrammatical expressions or turns including both. The frequencies were calculated in order to determine the proportion of the conversational speaking turns affected. Due to the small sample size, the durations and occurrences in turns per participant were examined and analysed using descriptive statistics (mean, median and range of variation) in order to compare the proportion of speech disfluencies in the ASD group with that of their age- and gender-matching control group.

As disfluencies, we have examined the following features in the group conversations: word searches, self-repairs, false starts, fillers, prolongations, inconsistent syntactic structures, blocks and grammatical errors. As grammatical errors we have considered features such as incorrect case endings, inconsistent syntactic structures, unclear references and erroneous lexical choices. Examples of the different types of disfluencies and grammatical errors taken into account when classifying the two groups in the data are given in Table 4. In the examples, the talk is depicted on three lines: The first line is the original utterance in Finnish, the second line offers the word-by-word gloss in English, and the third line provides the English translation.

**Table 4:**
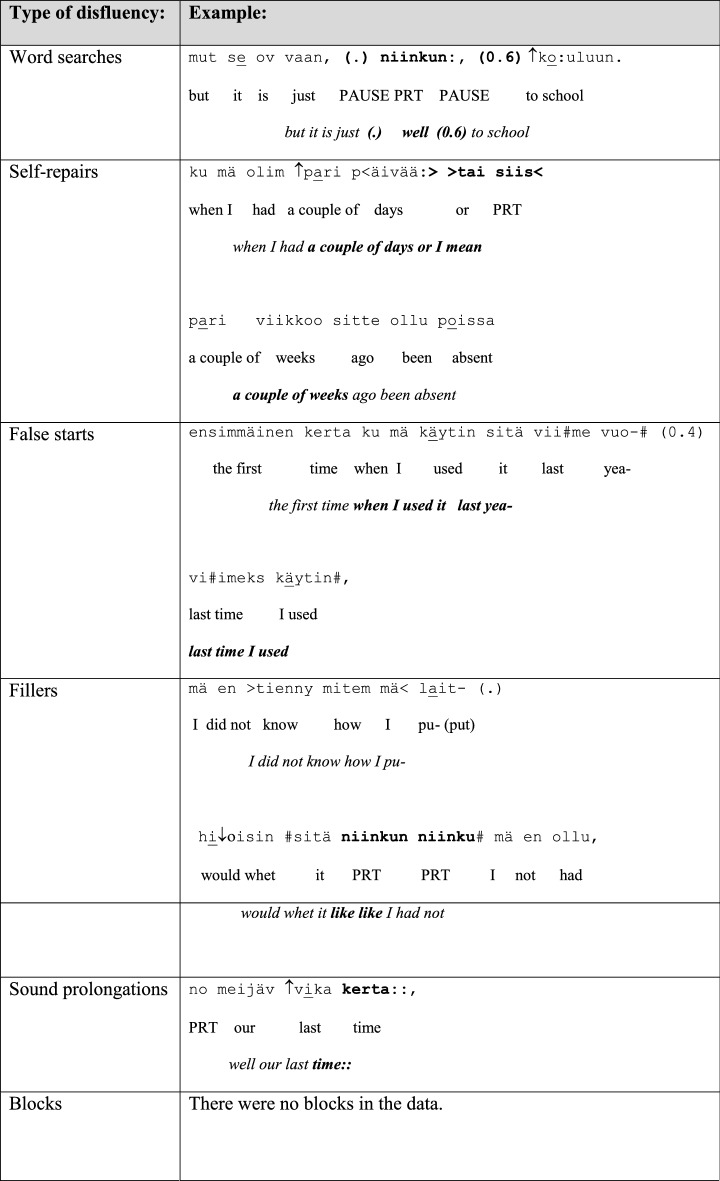

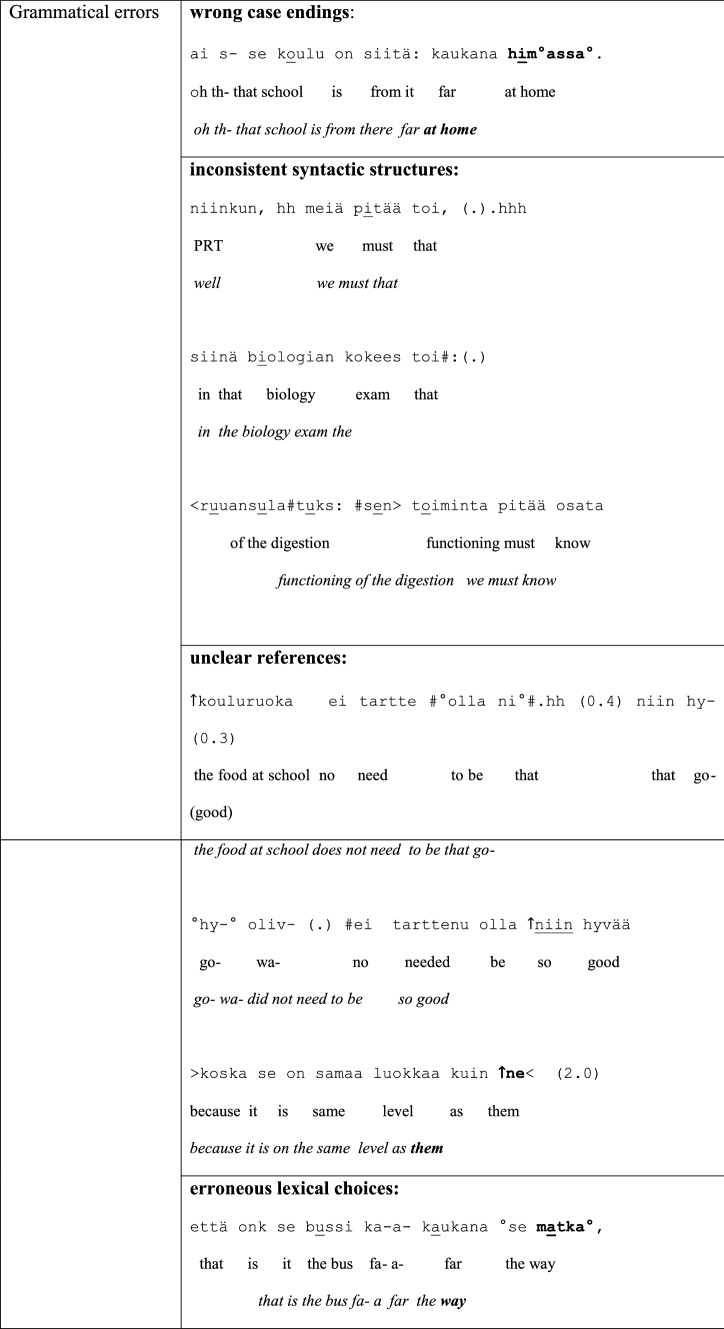
Different types of disfluencies and grammatical errors examined in this study

PRT = particle/interjection; (.) = micro pause; (0.6) = measured pause of 0.6 s; # = creaky voice; ↑ = rising shift in intonation; : = sound prolongation; <  >  = slow pace; >  <  = fast pace

For the qualitative analysis, the whole data were transcribed in the above manner by following the transcription conventions generally adopted within the framework of CA. The transcriptions aim at capturing not only *what* is being said, but also *how* something is being said, thus pauses were measured and features of speech production, such as creaky voice, sound prolongations and slowing and increasing pace, were marked (Hepburn and Bolden 2013, p. 57). Indeed, conversation analytic transcriptions are based on the assumption that “no order of detail in interaction can be dismissed a priori as disorderly, accidental, or irrelevant” (Heritage 1984, p. 241). The durations of pauses are indicated in brackets in tenths of a second (for example: (0.5) corresponds to 0.5 s), interrupted words are indicated with a hyphen (for example: *koir-*), and lengthened sounds are indicated with a colon (for example: *koiraa:*). The person and place names in the transcripts have been replaced with pseudonyms. A complete list of the signs and abbreviations used in transcribing the examples is provided in the Appendix. The aim of the qualitative analysis was to examine the types of disfluencies and ungrammatical expressions in more detail to observe how other participants in the interaction react to them. In order to detect the reactions and the interactional consequences of the atypical construction of conversational speaking turns, we analysed the interactional sequences consisting of interlocutors’ responses to speakers’ disfluent and/or ungrammatical turns.

## Results

### Proportions of Disfluencies and Ungrammatical Expressions in Conversational Speech by Preadolescents with ASD and Controls

**Fig. 1:**
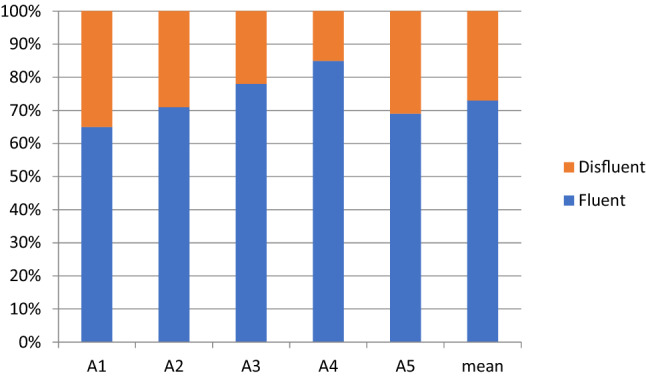
Percentages of fluent and disfluent speech of participants with ASD

The main result of our quantitative analyses was that the preadolescents with ASD produced more disfluencies and ungrammatical expressions than the controls. The mean proportion of disfluencies and ungrammatical expressions in the total duration of their speech was 26.4% for the ASD group and 15.5% for the control group. The group means of fluent and disfluent speech and distributions for each participant are presented in Figs. 1 (ASD group) and 2 (Control group).

**Fig. 2:**
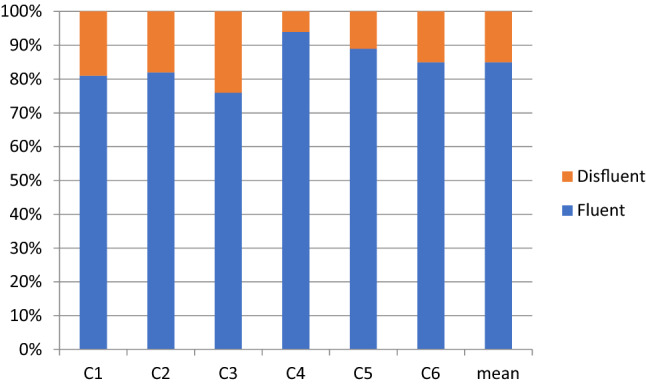
Percentages of fluent and disfluent speech of control participants

The disfluency percentages in the speech of the participants with ASD ranged from 35% by A1 to 15% by A4, with a mean of 26.4% and median of 29% (Fig. 1). In the control group, the range was from 24% by C3 to 6% by C4, with a mean of 15.5% and median of 15% (Fig. 2). Although the means and medians of the ASD and control groups clearly differed, considerable individual variation occurred in the percentage of disfluency within the groups.

**Fig. 3:**
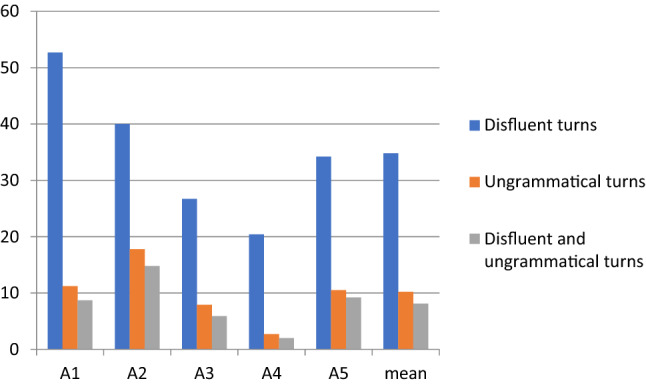
Percentual proportions of disfluent vs. ungrammatical speaking turns by ASD group

**Fig. 4:**
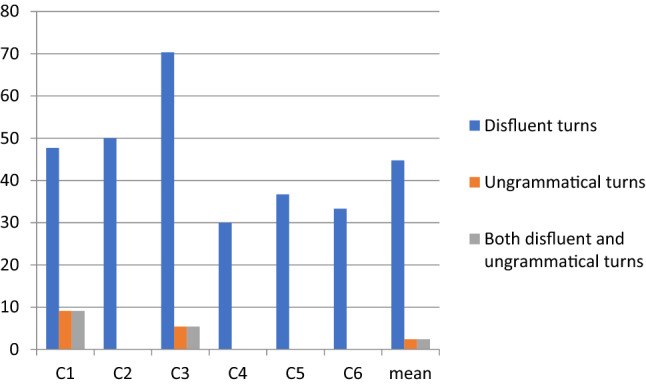
Percentual proportions of disfluent vs. ungrammatical speaking turns by control group

In total, ASD group produced more speaking turns (412 speaking turns in 30 min) than the control group (192 speaking turns in 30 min). When the number of conversational speaking turns affected by disfluency were examined, the mean proportion of disfluent speaking turns was greater in the control group (44.7%) than in the ASD group data (35%) (see Figs. 3 and 4). In the ASD group, the percentage of disfluent turns ranged from 52.7 to 20.4% with a median of 26.7%, whereas in the control group, the range was from 70.3 to 30% with a median of 42.2%. Although the controls produced more speaking turns with disfluencies, their disfluencies were mainly short pauses and single fillers or sound prolongations which did not disturb the overall flow of speaking.

In general, the fillers and sound prolongations in the speaking turns of the control group appeared as unproblematic short hesitations, whereas the disfluent speaking turns of preadolescents with ASD were more complex with word searches, self-repairs and false starts. Due to this complexity in turn construction, the mean proportion of speaking turns including ungrammatical expressions was greater in the ASD group (10.2%, see Fig. 3) than in the control group data (2%, see Fig. 4). In the group of preadolescents with ASD, the proportion of turns with ungrammatical expressions ranged from 18 to 3%. Only two out of six in the control group produced turns with ungrammatical expressions, and the percentages of ungrammatical turns were 9% and 5%. Furthermore, the speaking turns in the ASD group were more often both disfluent and ungrammatical than in the control group, which had speaking turns that were more often only disfluent.

### Qualitative Differences in Conversational Interaction Between ASD and Control Groups

In this section, we analyse the structure of the interaction in conversational sequences containing disfluent speech and ungrammatical expressions in our data corpus. The analyses illustrate in more detail the qualitative difference observed in the previous Sect. “Proportions of Disfluencies and Ungrammatical Expressions in Conversational Speech by Preadolescents with ASD and Controls” between the disfluencies and ungrammatical expressions occurring in the speaking turns of the participants with ASD and the control group. We first focus on the participants with ASD (3.2.1) and then on the participants of the control group (3.2.2).

### Disfluencies and Ungrammatical Expressions in Conversations of Participants with ASD

The first example occurs in the Group A session. In this extract, morpho-syntactic disfluency in speech production leads to a self-repair by the participant with ASD and the therapist’s intervention, that is, to therapist-supported problem solving.^5^ Just before this extract, one of the boys, Harri, has stated that he always takes the bus to school when the weather is not good. Another boy, Markus, reacts to this turn by stating that he also takes the bus (line 01). Harri becomes disfluent and displays morpho-syntactic problems in constructing his speaking turns in lines 8–9 and 12. Similarly, Markus displays disfluency and an ungrammatical construction during his speaking turns in lines 15–16 and 25–26. The speaking turns that constitute the focus of the analysis are marked with arrows.

#### Example 1:

Bus to school. Markus = A2, Harri = A1.


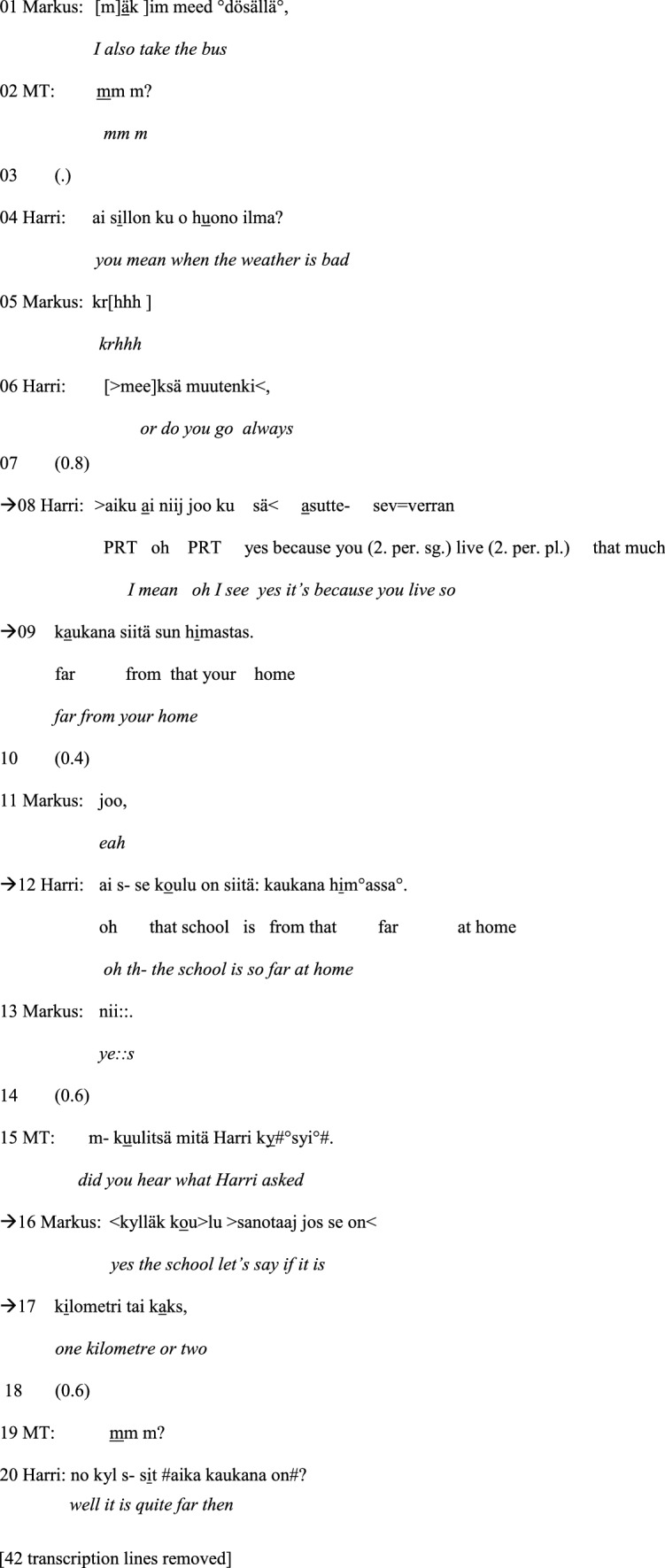

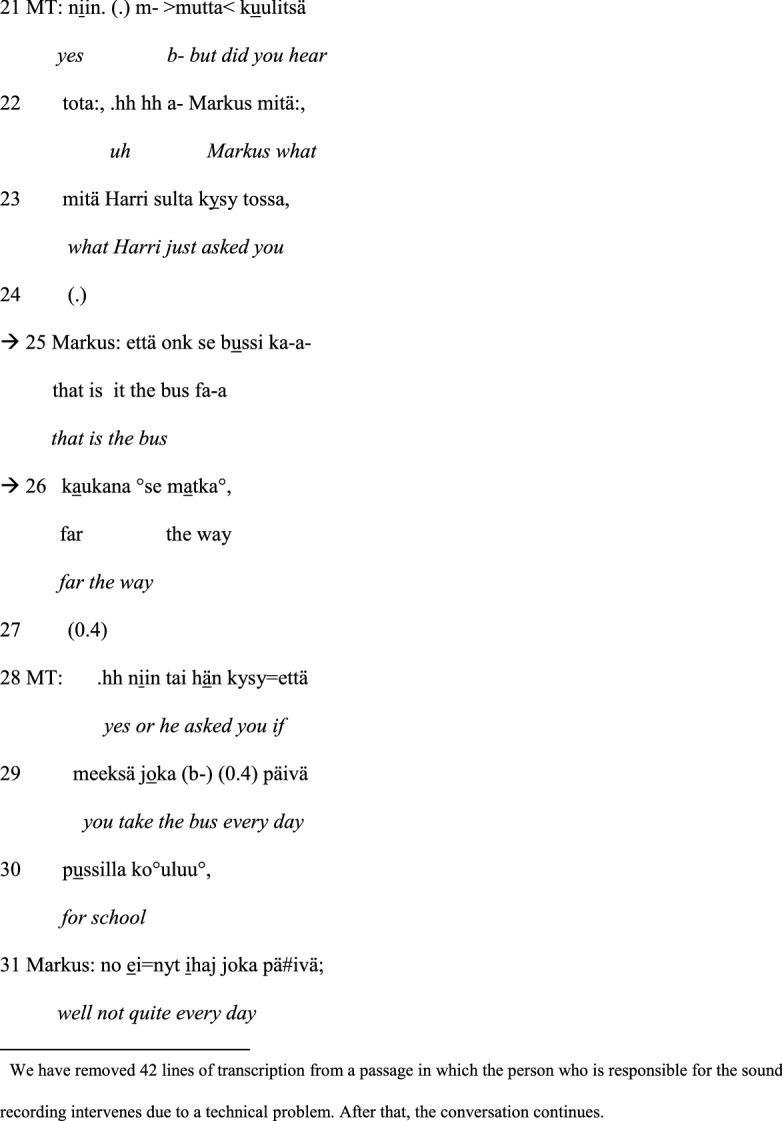


After Markus has stated that he also takes the bus to school, Harri asks Markus if he only takes the bus when the weather is not good (line 04) or otherwise as well (line 06). Markus does not immediately answer, and Harri subsequently continues with an ungrammatically constructed turn in which Harri seems to draw the conclusion that Markus lives so far from school that he has to take the bus (lines 08–09). The word ‘school’ (*koulu*) is, however, missing from Harri’s turn. Instead, the turn includes the expression ‘from your home’ (*himastas*)^6^ (line 09), which makes the turn incoherent (‘you live so far from your home’). Markus answers ‘yeah’ (*joo*) (line 11), which displays that he appears to have understood Harri’s question. Nevertheless, after that, Harri tries to reformulate his question addressing the distance between home and school (line 12). This time, the words ‘school’ (*koulu*) and ‘home’ (*hima*) are both included in the turn. The word *hima* (‘home’) has, however, the wrong case ending: Harri says *himassa*, which is in the inessive case and means ‘at home’, whereas a grammatically better suited expression would have been *himasta*, which is in the elative case and means ‘from home’. Markus responds with the discourse particle *nii::* (‘yes’) (line 13), which is lengthened. The lengthening of this particle could indicate that Markus has difficulty interpreting the previous turn and that he expects Harri to continue adding information to his turn. This conversational exchange shows how the ungrammatical construction of the speaking turn makes it difficult for the recipient to comprehend.

As it is uncertain whether the boys have understood each other, the male therapist intervenes and asks Markus whether he has understood Harri’s questions (line 15). In his answer, Markus reacts to Harri’s turn in lines 08–09, using a grammatically sparse expression referring to the distance of one or two kilometres (lines 16–17). Nevertheless, Harri seems to understand it and manages to formulate a coherent and a fluent turn: *no kyl s- sit aika kaukana on* (‘well it is quite far then’) (line 20). After the interruption caused by a technical problem, the male therapist returns to the earlier discussion by indirectly encouraging Markus to respond to the first questions Harri has asked in lines 04 and 06, of whether Markus always took the bus to school or only when the weather was not good, by asking whether Markus heard what Harri asked (lines 21–23). In recollecting what Harri had asked, Markus answers by referring to bus and the trip being far (lines 25–26). The structure of the turn is disfluent with cut-off sounds and is incoherent as he does not mention home and the school, i.e., which way/distance he means. Thus, at this point, Markus also has difficulties expressing himself. The male therapist decides to try to resolve the comprehension problem by offering his interpretation of Harri’s original question (lines 28–30). In fact, this resolves the problem, and Markus manages to formulate a fluent and a coherent turn as an answer (line 31).

Thus, to conclude, Example 1 illustrates how the preadolescents with ASD become disfluent and display difficulties in the grammatical and coherent construction of their speaking turns. This becomes particularly apparent during conversational sequences that consist of questions and answers, in which the intersubjective understanding between the participants is at stake. Due to the difficulties in forming and answering questions fluently, their conversational interaction is supported by the therapist who points out conversational sequences containing questions that have not been understood and answered. With their therapist’s support, the problems are, however, efficiently solved, and the participants with ASD are able to produce fluent speaking turns.

### Disfluencies and Ungrammatical Expressions in Conversations of the Control Group

As the quantitative analyses presented above demonstrated (cf. 3.1.), ungrammatical expressions were not frequent in the control group data. Only two members of the control group members had very few speaking turns that include grammatical problems, whereas all participants with ASD produced many ungrammatical speaking turns as well as turns that were both ungrammatical and disfluent. Thus, the example presented below of the control group’s typical speaking turns will only illustrate disfluencies and not ungrammatical expressions. Disfluencies that occur in the control group data are mainly fillers and sound prolongations, which do not cause comprehension problems (see Example 2). During this passage, the group is talking about their hobbies and free time, and one of the boys, Pekka, states that he does not have much free time due to his frequent training sessions and games (lines 01–03). The speaking turns in the focus of the analysis are marked with arrows and the disfluencies are indicated in bold face.

#### Example 2:

Football practice. Pekka = C3; Miikka = C5.


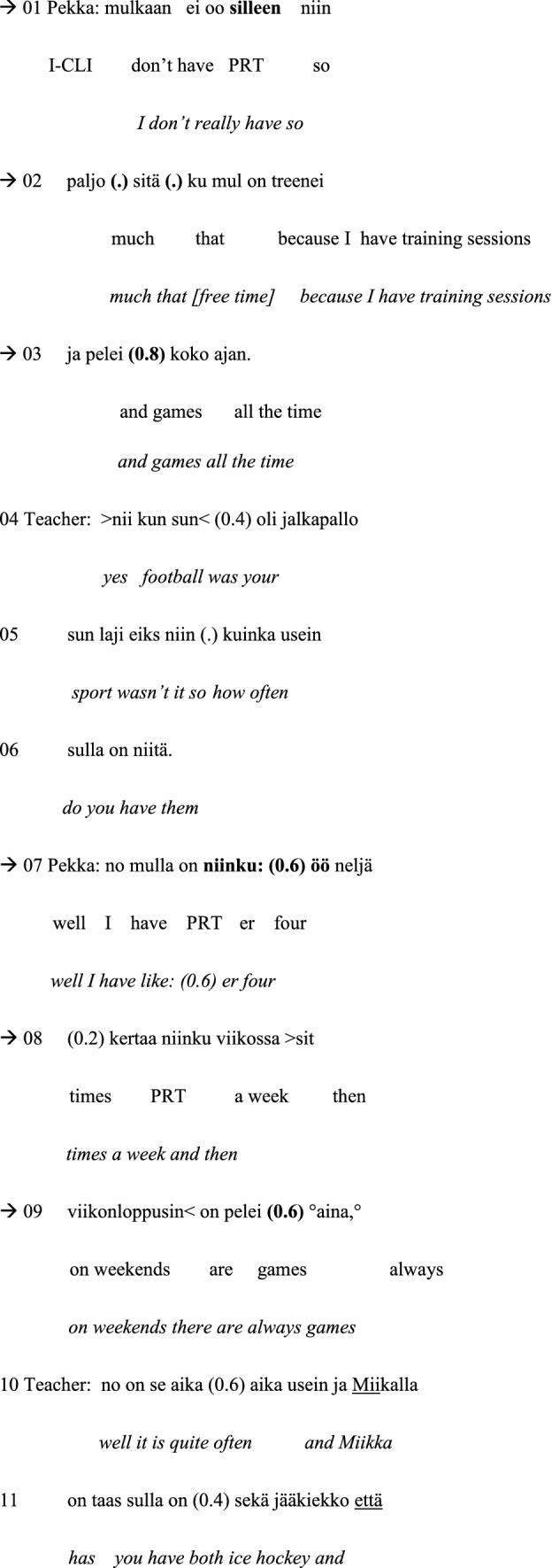

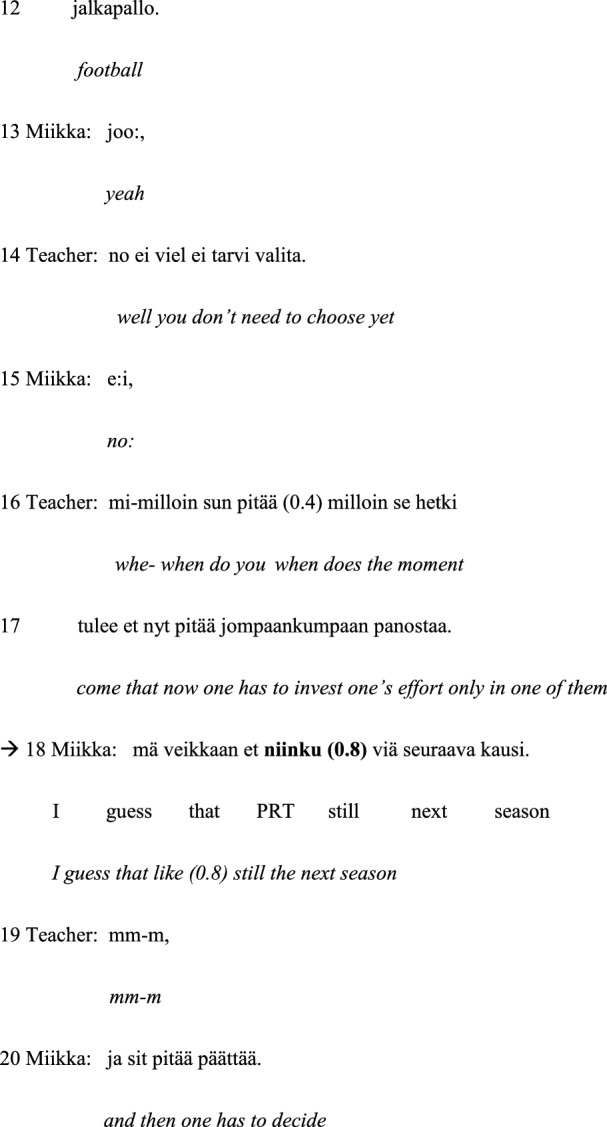


In this example, the speakers, Pekka and Miikka, produce some fillers and pauses. Pekka first uses the filler *silleen* (a particle which could be roughly translated as ‘that way’, line 01). This filler is typically related to speech planning (Hakulinen et al. 2004, § 861) and also occurs here in a syntactically well-positioned place. The general impression of his turn is that it is fluent, with no cut-off utterances, such as searching, revision or restarting, and the occurrence of the filler and short pauses do not lead to comprehension problems. On the contrary, the teacher reacts to Pekka’s turn with a follow-up question (lines 04–06) the contents of which demonstrate that she has understood the preceding turn: As the boy has said that he does not have much free time due to his training sessions and games (lines 01–03), the teacher asks him how often he has them.

During his second turn (lines 07–09), Pekka first produces the filler *niinku:* (a particle which could be roughly translated as ‘like’ or ‘kind of’), which is prolonged (line 07). This filler is also typically related to the planning of speech (Hakulinen et al. 2004, § 861). Thus, the prolongation that it carries, as well as the pause (0.6 s) and vocalization *öö* (‘er’), occurring after it, emphasize this function. In addition, here the filler and vocalization are syntactically well positioned to display to the listener that the speaker is thinking continuing his utterance, thus enabling him to maintain his speaking turn during planning. Shortly thereafter (line 08), there is yet another occurrence of the *niinku* filler, which reflects similar speech planning. These fillers do not create any comprehension problems: The contents of the beginning of the turn produced by the teacher (*no on se aika usein*, ‘well it is quite often’, line 10) indicate that she has understood the preceding turn.

The teacher then addresses another participant, Miikka (lines 10–12) by stating that Miikka plays both ice hockey and football. The boy answers affirmatively with *joo* (‘yeah’) (line 13). The teacher states that Miikka does not yet need to choose between the two sports (line 14), and Miikka answers *e:i*, (‘no:’) (line 15). After that, the teacher asks Miikka when he has to make the choice between these two sports (lines 16–17). The boy answers *mä veikkaan et niinku (0.8) viä seuraava kausi* (‘I guess that like (pause) still the next season’, line 18). This turn includes an occurrence of the filler particle *niinku* that is followed by a rather long (0.8 s) pause, and both indicate speech planning (Hakulinen et al. 2004, § 861). The filler and the pause do not cause comprehension problems in this context: The teacher produces a turn consisting of the particle *mm-m* (‘mm-m’, line 19), which indicates that she is listening (Hakulinen et al. 2004, § 798), after which the boy continues *ja sit pitää päättää* (‘and then one has to decide’, line 20). To conclude, Example 2 illustrates how the preadolescents of the control group use some filler particles and pauses while planning their speech, but their speaking turns are nevertheless fluent and easily understood, and they do not need support from their teacher to answer the questions coherently.

## Discussion

In our study, the durational mean proportions of disfluencies and ungrammatical expressions were greater in the speech of participants with high-functioning ASD/AS (26.4%) than in the control group (15.5%). Thus, our study confirms the findings by Lake et al. (2011), Scaler Scott et al. (2014) and Shriberg et al. (2001) that more disfluencies occur in the speech of individuals with ASD than in the speech of matched controls. Individual variation in the amount of disfluency was high within both groups, which is in line with the results reported on large individual ranges of speech disfluency in adult speakers (e.g., McDougall and Duckworth 2017). Furthermore, a qualitative difference was noted: The ASD group produced long and complex disfluent turns with word searches, self-repairs, false starts, fillers, sound prolongations, inconsistent syntactic structures and grammatical errors, whereas the control group mainly had disfluencies that were fillers and sound prolongations. Previously, a qualitative difference has been reported by Lake et al. (2011), who demonstrated that adult speakers with AS produced fewer listener-oriented disfluencies such as filled pauses and revisions and more silent pauses and repetitions that reflect the speaker’s speech processing. This result is in line with our findings in the sense that the disfluencies produced by the speakers with AS in our study also seem to reflect the speaker’s own grammatically disturbed speech processing, whereas the disfluencies (fillers and sound prolongations) produced by the control group members were more listener-oriented. When the percentages of speaking turns with disfluencies were compared, control speakers produced more disfluent speaking turns with one or two mild disfluencies such as a filler particle *niinku* (appr. “like”) and the vocalization *öö* (“er” or “um”) than preadolescents with ASD, who produced more speaking turns with grammatical errors and complex disfluencies. A qualitative difference in the types of disfluencies have also been observed previously between 4- and 8-year-old children with ASD and their age-matched controls: Control children produced more fillers (e.g. *um, uh*) (MacFarlane et al. 2017). It is also important to note that disfluencies and grammatical incoherence tended to co-occur in the speech of preadolescents with ASD, whereas similar co-occurrences were not observed in the control group.

In addition, due to the quantitative and qualitative difference, the disfluencies and the ungrammatical expressions that arose in the ASD participants’ interactions also caused comprehension problems during conversational interaction (see also Wiklund and Laakso 2019), whereas the control group did not experience comprehension problems. The control group’s disfluencies were similar to the hesitations that normally fluent speakers (c.f., McDougall and Duckworth 2017) produce in order to plan expressions and to help listeners project that a continuation is to come. The participants with ASD often had a combination of disfluencies and grammatical errors and this caused more profound problems for the listeners to understand what was said. As a consequence, the disfluency of participants with ASD had more serious effects for the interactional flow of their conversations in comparison to the age- and gender-matched controls. Our study points out that it would be very important to study in a more extensive manner the conversational interactions of participants with ASD. Further studies may reveal how the interactional difficulties arise from the morpho-syntactic level of constructing coherent speaking turns and finding correct lexical items, challenging the view of autism as a disorder of the pragmatic use of language. In the future, it would also be important to study mixed groups including participants with and without ASD in order to examine if the quantitative and qualitative difference discovered in this study can also be found in mixed groups.

The disfluencies produced by preadolescents with ASD in our data were predominantly more complex than mere repetitions. This finding is in contrast with some prior studies that have reported syllable, word or phrase repetitions that occur frequently in the speech of adult speakers with AS (e.g., Lake et al. 2011; Scott et al. 2006; Shriberg et al. 2001; Sisskin 2006). Instead, in the current study, the disfluencies by the preadolescents with ASD appeared to result from their problems in constructing grammatically coherent utterances, and displaying a difficulty in finding and/or selecting correct lexical items, both words and case endings. This difference as compared to prior studies of adults may reflect the age of the participants (i.e., the lexical and grammatical abilities by the preadolescents with ASD were not yet fully developed) or the grammatical structure of the highly inflected language, Finnish, which the preadolescents in our data were speaking. To examine the latter, it would be necessary to conduct comparative studies between data from different languages, and this is one direction for future research. In addition, the age and development of lexical and grammatical abilities may play a role, as some previous studies on disfluencies by children with ASD (e.g., MacFarlane et al. 2017; Plexico et al. 2010) have reported that English-speaking children with ASD produce atypical disfluencies related to revising the content of speech instead of plain fillers, which are produced by children with typical development. Atypical disfluencies are not observed in children with developmental stuttering either. The comparison of our results to previous studies is however difficult because the definitions and terminology related to disfluency differs and the grammatical construction of utterances is seldom examined at the same time. In future studies, it would be interesting to count the numbers of occurrences of different types of disfluencies and ungrammatical utterances in detail and to compare ASD and control groups from this point of view in larger data sets allowing statistical analyses. In particular, our study suggests that it would be important to conduct longitudinal studies or cross-sectional studies of different age groups of children with ASD in which both the lexico-grammatical development and disfluency features would be examined.

The speech of the participants with ASD in our study was characterised by morpho-syntactic problems such as incorrect case endings, ambiguous pronominal references, disconnected syntactic structures and verb tense problems. These difficulties in constructing fluent utterances can also be connected to the previous findings of problems in higher-level cognitive processes such as attention, working memory, and executive function, which have been observed by individuals with high-functioning autism (e.g Joseph et al. 2005; Kenworthy et al. 2008). Thus, future research could also address the connections between these cognitive abilities and the lexico-grammatical construction of conversational speech by individuals with ASD.

As our data constitutes authentic interaction, we believe that the results also apply at least in a part to other types of spontaneous conversation. However, in the future, it would be essential to study disfluencies, grammatical problems and their interactional consequences by individuals with ASD in different types of everyday conversational interactions. The current multi-case study can be seen as a pilot that will hopefully be followed up in the future by research based on more data from everyday social encounters of a larger group of participants with and without ASD.

## Abbreviations

CLI: Clitic
PER: Person
PL: Plural
PRT: Particle
SG: Singular

## List of Symbols

.: Strongly falling pitch at the end of a prosodic unit
;: Slightly falling pitch at the end of a prosodic unit
,: Flat pitch at the end of a prosodic unit
,?: Slightly rising pitch at the end of a prosodic unit
?: Strongly rising pitch at the end of a prosodic unit
↓: Segment produced on a lower pitch level than the surrounding speech
↑: Segment produced on a higher pitch level than the surrounding speech
sika: Prominent stress
> tosi <: Accelerated speech rate
< paitsi >: Slowed speech rate
joo: Lengthened vowel
MITÄ: Increased level of loudness
.hhh: Clearly audible inhalation (one ‘h’ corresponds to 0.1 s)
hhh: Clearly audible exhalation (one ‘h’ corresponds to 0.1 s)
.joo: Word produced with an inhalation
@just@: Marked voice
k(h)iva: Word produced laughingly
£niimpä£: Word produced smilingly
∙nii∙: Word produced more quietly than the surrounding speech
[: Overlap of speech begins
]: Overlap of speech ends
(.): Micropause (duration of less than 0.2 s)
(0.6): Pause (duration measured in seconds)
(lapset): Unclear speech
** = **: Turn starts immediately after the end of the previous turn

## Footnotes

1 Word-final disfluencies were defined in this study as part-word repetitions of final sounds or syllables of words (e.g. ‘chair-air’, ‘light-t’) or prolongations of the final sounds of words (e.g. ‘thissss’).

2 The total duration of each group therapy session was 2 h, but only 1 h is included in our data.

3 One of the characteristics of ASD is the lack of eagerness to share interests, joys and achievements with others and to display interest in other people’s preoccupations (APA 2013). Reciprocal social interaction in general is difficult for people with ASD. For these reasons, sharing news and asking questions are practiced in therapy groups.

4 The data collection of our experimental groups was completed in 2009 and 2010. However, the control group data were not collected before 2016. This time gap could possibly affect certain linguistic phenomena, because the language obviously changes over time. Nevertheless, we argue that six years is not long enough to have a significant effect in the phenomena under consideration.

5 Therapist-supported problem-solving has been analysed in Wiklund and Laakso (2019). This previous study investigates ASD persons’ disfluencies from a purely qualitative perspective, without any quantitative information and without comparing the findings to control group data.

6 *Hima* is a slang word that means ‘home’.
